# Expression and methylation status of tissue factor pathway inhibitor-2 gene in non-small-cell lung cancer

**DOI:** 10.1038/sj.bjc.6602298

**Published:** 2005-02-01

**Authors:** J Rollin, S Iochmann, C Bléchet, F Hubé, S Régina, S Guyétant, E Lemarié, P Reverdiau, Y Gruel

**Affiliations:** 1 INSERM U 618 ‘Protéases et Vectorisation Pulmonaires’ and IFR 135 Faculté de Médecine, 2 bis Bd Tonnellé, 37032 Tours Cedex, France

**Keywords:** TFPI-2, NSCLC, promoter hypermethylation, real-time PCR

## Abstract

Tissue factor pathway inhibitor-2 (TFPI-2) is a Kunitz-type serine proteinase inhibitor that inhibits plasmin-dependent activation of several metalloproteinases. Downregulation of TFPI-2 could thus enhance the invasive potential of neoplastic cells in several cancers, including lung cancer. In this study, TFPI-2 mRNA was measured using a real-time PCR method in tumours of 59 patients with non-small-cell lung cancer (NSCLC). Tumour TFPI-2 mRNA levels appeared well correlated with protein expression evaluated by immunohistochemistry and were 4–120 times lower compared to those of nonaffected lung tissue in 22 cases (37%). Hypermethylation of the TFPI-2 gene promoter was demonstrated by restriction enzyme-polymerase chain reaction in 12 of 40 cases of NSCLC (30%), including nine of 17 for whom tumour TFPI-2 gene expression was lower than in noncancerous tissue. In contrast, this epigenetic modification was shown in only three of 23 tumours in which no decrease in TFPI-2 synthesis was found (*P*=0.016). Decreased TFPI-2 gene expression and hypermethylation were more frequently associated with stages III or IV NSCLC (eight out of 10, *P*=0.02) and the TFPI-2 gene promoter was more frequently hypermethylated in patients with lymph node metastases (eight out of 16, *P*=0.02). These results suggest that silencing of the TFPI-2 gene by hypermethylation might contribute to tumour progression in NSCLC.

Lung cancer is a major cause of death throughout the world and develops slowly over many years from sequential accumulation of multiple gene changes in pulmonary cells (Zochbauer-Muller *et al*, 2002). The major histological types of malignant lung tumours that affect about 80% of patients are non-small-cell lung cancers (NSCLC), which are divided into squamous cell carcinoma, adenocarcinoma and mixed types (Travis *et al*, 1999). When lung cancer is diagnosed at a localised stage, the 5-year survival is about 50%. In contrast, its prognosis is very poor when diagnosed with lymph node involvement or metastases, and the 5-year survival is then 8 and 3%, respectively (Etzioni *et al*, 2003). Several mechanisms are involved in the spread of metastatic cancer cells from the primary tumour, and degradation of the extracellular matrix (ECM) is an important process involving a variety of matrix-degrading proteinases. The most important proteolytic ECM enzymes are metalloproteinases (MMPs), and upregulation of MMPs expression has been reported to be strongly associated with the progression of malignancy in several types of cancer, including NSCLC (Cox *et al*, 1999; Egeblad and Werb, 2002). Metalloproteinases are zinc-dependent endopeptidases secreted as inactive proenzymes and some require activation by plasmin, a serine proteinase. One potent inhibitor of plasmin within the ECM is tissue factor pathway inhibitor-2 (TFPI-2), also called matrix-associated serine protease inhibitor (MSPI). TFPI-2 is a 32 kDa Kunitz-type serine proteinase inhibitor also previously shown to reduce tumour invasion (Konduri *et al*, 2000, 2001). By inhibiting plasmin, TFPI-2 effectively decreases activation of MMP-1, MMP-3 and MMP-9 (Rao *et al*, 1999), and reduces the invasive potential of several tumour cell lines including A549, an NSCLC cell line (Lakka *et al*, 2000). The human TFPI-2 gene is located on chromosome 7q22 (Miyagi *et al*, 1996) and its promoter contains a CpG island region of at least 220 bp that spans exon 1 and the three transcription initiation sites. With a G+C content of about 77% and an observed/expected presence of CpG>0.85, this region also coincides with the 5' end of the minimal promoter (Hube *et al*, 2003a). CpG islands in normal cells are protected from methylation by mechanisms that are poorly understood. In contrast, hypermethylation of CpG islands in the promoter region of tumour suppressor genes is a well-known mechanism of gene silencing that contributes to tumour progression in many cancers (Herman and Baylin, 2003). Transcriptional silencing of the TFPI-2 gene by promoter hypermethylation was recently demonstrated in human glioma cells and choriocarcinoma cells (Hube *et al*, 2003b; Konduri *et al*, 2003). The TFPI-2 gene might therefore be a candidate tumour suppressor gene.

In this study, we first investigated TFPI-2 expression in several histological types of NSCLC by a specific real-time polymerase chain reaction (PCR) method. We then studied the methylation status of human TFPI-2 promoter gene in a consecutive series of lung tumours using methyl-sensitive restriction enzyme and bisulfite genomic sequencing methods to evaluate whether hypermethylation of the promoter gene might be responsible for TFPI-2 gene downregulation.

## MATERIALS AND METHODS

### Patients and samples

We analysed tissue samples collected in a prospective series of 59 patients with NSCLC who had undergone complete surgical resection of the lung tumour as initial treatment (i.e. without prior radiotherapy or chemotherapy) between January 2002 and May 2003 in our hospital (CHU Trousseau Tours, France). Tumoral and nontumoral tissue samples were selected by a pathologist from fresh surgical specimens from every patient, and immediately stored in RNA*later* (Ambion, Austin, TX, USA) until RNA or DNA extraction and further analysis.

Histological diagnosis and grade of differentiation were determined in accordance with the World Health Organization (WHO) criteria for lung tumours (Travis *et al*, 1999) and pathologic stage was based on the revised international system (Mountain, 1997).

### RNA extraction and cDNA synthesis

Total RNA was extracted from samples using the perfect RNA Mini Kit (Eppendorf, Hambourg, Germany) according to the manufacturer's instructions. The disruption and homogenisation of tissue samples were first performed by grinding to a powder in liquid nitrogen. Samples were then solubilised using a chaotropic guanidinium isothiocyanate solution. The lysates were incubated with an RNA-binding matrix, and finally total RNA was eluted after washing with spin column chromatography. RNA yield and purity were determined by spectrophotometry and only samples with an A_260_/A_280_ ratio above 1.6 were kept for further experiments. Four micrograms of total RNA were then reverse-transcribed for 1 h at 42°C in 1 × buffer containing 125 *μ*M of each deoxynucleotide triphosphate, 2.5 *μ*M random decamers (Ambion), 25 U of RNase inhibitor and 20 U of AMV Reverse Transcriptase (Roche Diagnostics, Meylan, France).

### Real-time quantitative PCR assay for TFPI-2 mRNA

The amount of TFPI-2 transcripts within lung tumours and nonaffected tissues was assessed by real-time PCR using the icycler iQ detection system (Bio-Rad, Ivry sur Seine, France). The primers used to study TFPI-2 gene transcription were defined taking into account the similarities between TFPI-1 and TFPI-2 and to avoid hybridisation to homologous sequences (Table 1). Transcripts specific for the gene encoding 18S ribosomal RNA were also quantified for all samples using previously designed primers (Wang *et al*, 2002). This gene was therefore used as an internal control to evaluate the amount and quality of cDNA in every sample.

Every TFPI-2-specific PCR was performed in a total reaction volume of 25 *μ*l containing cDNA obtained from 100 ng of total RNA, 1 × of Platinum Quantitative PCR SuperMix-UDG (Invitrogen™, Cergy Pontoise, France), 0.32 *μ*M of each primer (Proligo, Paris, France) and 0.2 × of Sybr Green solution (Roche). To study TFPI-2 gene expression, the PCR was initiated by a decontamination (50°C for 2 min) and denaturation step (95°C, 2 min), followed by 35 cycles at 95°C for 20 s and at 67°C for 40 s. The PCR conditions to quantify the amount of 18S transcripts were different with regard to the amount of cDNA template (5 ng), the primer concentration (0.64 mM), and 25 cycles (95°C for 20 s, and 65°C for 40 s) were performed. The melting curve was analysed for each sample to check PCR specificity.

In order to express our results, specific standard curves were established using purified PCR products of 750 bp for TFPI-2 and 850 bp for 18S. Decreasing amounts of these DNA fragments (from 2.5 × 10^7^ to 2.5 × 10^1^ copies and 2 × 10^8^ to 2 × 10^5^ copies for TFPI-2 and 18S genes, respectively) were used for this purpose.

Each sample from either nonaffected lung or tumour tissue was studied in duplicate and the *C*_t_ value obtained (threshold cycle) allowed determination of the amount of the starting target message using the specific standard curve.

The results for each sample (noncancerous or tumour tissue) were expressed as the number of TFPI-2 mRNA copies for 10^7^ copies of 18S. In addition, the ‘*N*_TFPI-2_’ value, that reflects TFPI-2 gene expression within a tumour compared to that of the corresponding nonaffected (NA) lung, was calculated for each patient as follows: 
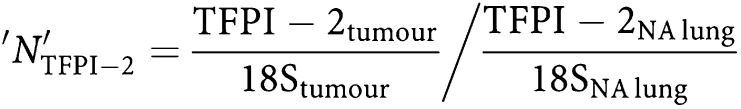


### Immunohistochemical localisation of TFPI-2

Formalin-fixed, paraffin-embedded samples of noncancerous pulmonary tissue and lung tumour were studied for 22 patients. In brief, 4-*μ*m-thick tissue sections were deparaffinised, rehydrated, immersed in EDTA buffer and subjected to antigen microwave retrieval. The primary antibody was a polyclonal (rabbit) anti-human TFPI-2 IgG (Rao *et al*, 1996) used at 1/1000 dilution. Immunostaining for TFPI-2 was then revealed using a standard streptavidin–biotin peroxidase complex (LSAB) method with diaminobenzidine as a chromogen (ChemMate™ Detection kit, DakoCytomation, Denmark). Mature placenta tissue known to express large amounts of TFPI-2 was used as a positive control, with haematin counterstaining. Two independent observers evaluated the topography and intensity of the staining. Staining scores of stroma cells and tumour cells (ranging from 0 to 300) were established by multiplying the staining intensity scaled from 0 to 3 (0=null, 1=weak, 2=moderate, and 3=strong) by the percentage of positive cells.

### Dot blot analysis

Dot blot analysis was performed for 12 tumour samples expressing either low (*n*=6) or high (*n*=6) levels of TFPI-2 mRNA. Tissue samples were ground in liquid nitrogen, homogenised in 1 ml of TNC buffer (50 mM Tris-HCl, 0.15 mM NaCl, 10 mM CaCl_2_, 0.02% NaN_3_, 0.05% Brij35), and then centrifuged at 15 000 r.p.m. for 15 min. The protein concentration of each supernatant was measured by Lowry's method (Total Protein Kit, Sigma, Saint Quentin Fallavier, France). A total of 2.5 *μ*g of total proteins (20 *μ*l) were spotted on a nitrocellulose membrane and dried overnight. The membrane was incubated for 1 h with anti-TFPI-2 antibody diluted 1/3000 in TNT buffer (100 mM Tris HCl; 1.5 NaCl, 0.5% Tween 20) and for 1 h with peroxidase-labelled anti-rabbit IgG (Sigma) after washing with TNT. Following exposure for 1 min to the Chemiluminescence Reagent Plus (Perkin-Elmer Biosystems, Courtaboeuf, France), the membrane was drained, wrapped in Saran-Wrap and exposed to autoradiography film (Kodak, NY, USA) for 1–2 min in the dark. For each experiment, increasing amounts of protein (0.0625, 0.125, 0.25, 0.5 and 1 *μ*g) extracted from NCI-H23 (NSCLC cell line), previously identified as strongly expressing TFPI-2, were systematically tested. A standard curve was established defining that 1 *μ*g of NCI-H23 proteins contained 10 arbitrary units (AU) of TFPI-2. Results were then expressed in AU per microgram of protein after measurement of spot intensities by the Multi Analyst/Macintosh software (Biorad).

### Methylation analysis of TFPI-2 promoter

To determine the methylation status of the TFPI-2 promoter, a restriction enzyme-related polymerase chain reaction (RE-PCR) method was first applied to the study of samples collected from 40 patients for whom remaining material was available. Preparation of genomic DNA from tumour and nonaffected lung samples was performed using a DNA purification kit (Qiagen, Courtaboeuf, France). Every sample was lysed using proteinase K. DNA in the lysate was then bound to a silica-gel membrane and eluted in water after washing. Genomic DNA (4 *μ*g) was then predigested with 40 U H*ind*III (Promega, Lyon, France) overnight at 37°C and 1 *μ*g of the restricted DNA was incubated for 16 h at 37°C with 10 U E*ag*I or 5 U H*ga*I (Ozyme, Saint Quentin Yvelines, France). For the PCR, all restriction endonuclease-treated samples were diluted 1 : 20 in water. PCR was performed in a 25 *μ*l reaction containing 10 mM Tris-HCl pH 9.0, 50 mM KCl, 0.01% (w v^−1^) gelatin, 1.5 mM MgCl_2_, 0.1% Triton X-100, 50 *μ*M of each deoxynucleotide triphosphate, 0.5 U of Super *Taq* DNA polymerase (ATGC Biotechnologies, Noisy Le Grand, France), and 1 *μ*M of forward and reverse synthesised oligonucleotide primers (Table 1) (Proligo). GC-rich solution (Roche Diagnostics) was also used to facilitate amplification of GC-rich sequences. PCR was set up in a GeneAmp PCR system 2400 (Applied Biosystems, Courtaboeuf, France) with a touchdown programme to increase the specificity. The temperature profile was 2 min at 95°C followed by amplification for 20 cycles with a 0.5°C decrease in the annealing temperature after each cycle. The starting cycle consisted of 30 s at 95°C, 30 s at 70°C, and 30 s at 72°C. In total, 20 additional cycles were performed, with a fixed annealing temperature of 60°C, and a final extension for 10 min at 72°C. PCR products were then analysed by electrophoresis through 1.6% agarose gel in TBE buffer (90 mM Tris-HCl, 90 mM borate acid, 2.5 mM EDTA) containing 1 *μ*g ml^−1^ ethidium bromide. Finally, PCR products were visualised by UV trans-illumination (Gel Doc 1000 system, Bio Rad) and band intensities were measured (Multi analyst/Macintosh software). The TFPI-2 gene promoter was considered as hypermethylated within the tumour when the band intensity measured after RE-PCR performed with DNA extracted from the cancerous biopsy was at least twice as high as those obtained after studying DNA isolated from the nonaffected lung.

The methylation status of each CpG dinucleotide within a 250 bp region (−207/48 from translation start site) of the TFPI-2 promoter was also studied by bisulphite genomic sequencing in seven patients selected as described below according to the results obtained by real-time PCR (*N*_TFPI-2_ values) and RE-PCR. Genomic DNA was converted by sodium bisulphite as recently described by Hube *et al* (2003b). Briefly, 4 *μ*g of DNA were digested overnight with 5 U *Hind*III and then denatured with 0.3 M NaOH. Sodium bisulphite solution was added at a final concentration of 3 M and incubated for 18 h at 55°C, pH 5.0, in the dark with 20 mM hydroquinone. DNA was desalted with the GFX PCR DNA and gel purification Kit (Amersham Pharmacia Biotech Europe GMBH, Paris, France) and then desulphonated with 0.3 M NaOH before being precipitated by ethanol. Finally, DNA was amplified using nested PCR as previously described (Hube *et al*, 2003b) and the products obtained were then purified and cloned into pCR 2.1 TOPO vectors (Invitrogen). Three clones were selected after each experiment and sequenced to determine the methylation status in every sample. Choriocarcinoma JAR cells and trophoblast cells isolated from normal placentas (Hube *et al*, 2003c) were also studied as positive (i.e. hypermethylated) and negative controls for every experiment (RE-PCR or bisulphite genomic sequencing).

### Statistical analysis

The Sign test was used to evaluate the distribution of TFPI-2 mRNA levels in nonaffected lung tissues and tumour samples. The staining scores of immunohistochemistry were compared using the Mann–Whitney *U*-test. Analyses of the association between TFPI-2 mRNA expression, gene methylation status and clinicopathological feactures were performed with Pearson's *χ*^2^. All *P*-values obtained were considered as significant when ⩽0.05.

## RESULTS

### TFPI-2 mRNA expression in NSCLC

The real-time PCR developed to study TFPI-2 gene expression was first evaluated with varying amounts of a 750-bp TFPI-2 cDNA used as template. A strong linear relationship between the *C*_t_ and the log of the number of copies was consistently demonstrated (*R*^2^⩾0.99). The efficiency of the reaction ranged from 92 to 97%, with low intra- and inter-assay variations (0.8 and 2.3%, respectively). Similar results were recorded with the real-time PCR developed to study the 18S gene (efficiency 95.7% with intra- and inter-assay variations of 1.4 and 2.4% respectively).

**Figure 1:**
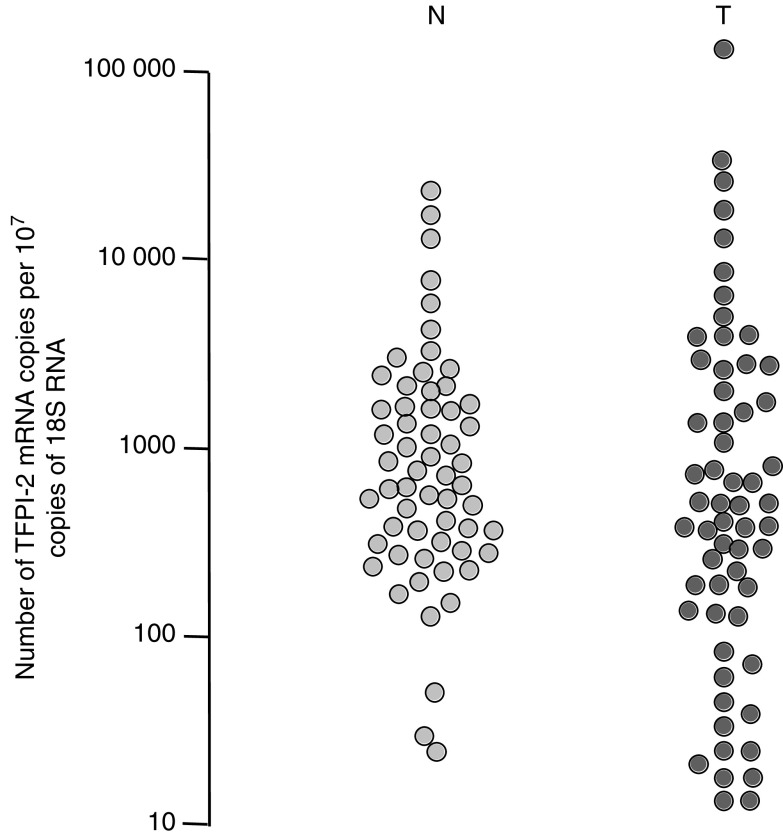
TFPI-2 gene expression in nonaffected lung tissue (N) and non-small-cell lung tumours (T). Each point is representative of at least two duplicates.

**Figure 2:**
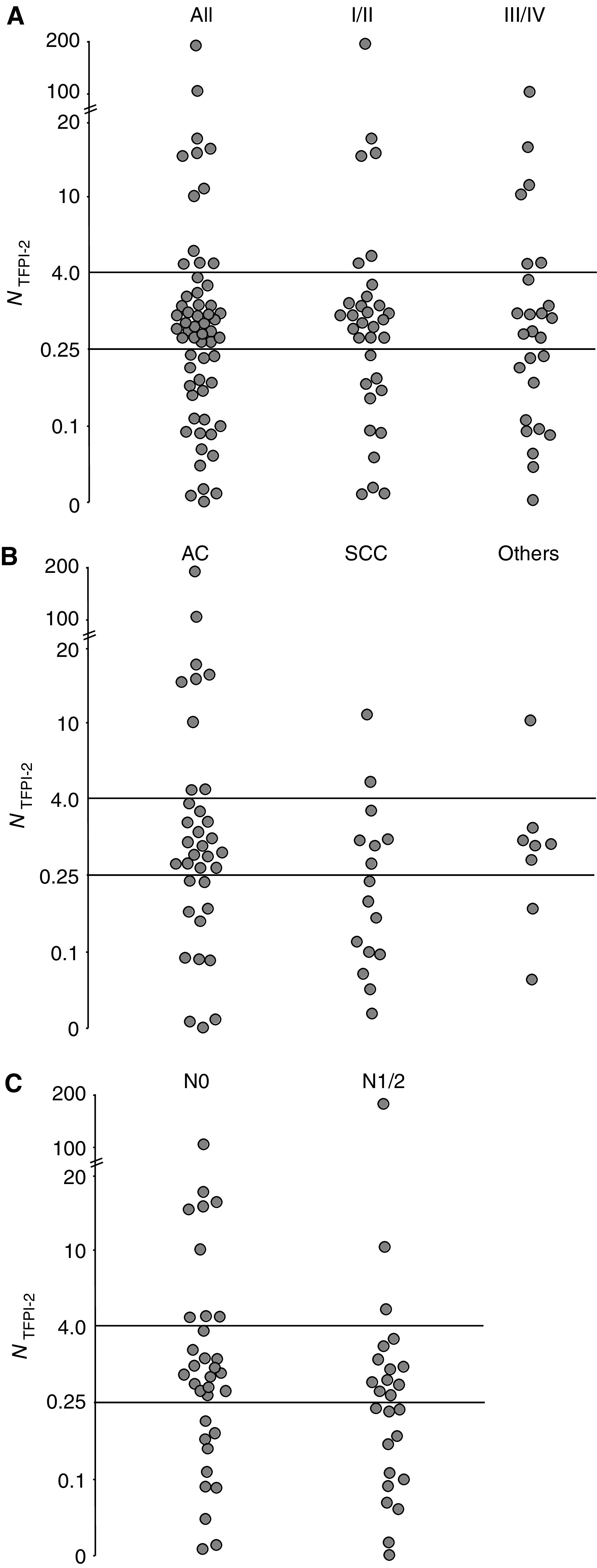
Relative TFPI-2 gene expression in NSCL tumours. The ‘*N*_TFPI-2_’ values are shown according to the stage of the disease (panel **A**), the histological type of the lung cancer: AC=adenocarcinoma; SCC=squamous cell carcinoma; others=other histological subtypes (panel **B**), and the presence (N1–N2) or absence (NO) of lymph node metastases (panel **C**).

Highly variable amounts of TFPI-2 mRNA were measured within both the nonaffected lung (32–20 400 copies/10^7^ 18S RNA) and the tumour (15–336 000 copies) and the distribution of values obtained was similar in both categories of tissue (*P*=0.5) (Figure 1). Four samples (i.e. two from the nonaffected lung and two from the tumour) were analysed for 25 patients, and the number of TFPI-2 mRNA copies measured within two biopsies obtained from the same tissue never varied more than four-fold (mean=1.8, and range=1.1–3.9). The amount of TFPI-2 mRNA within the tumour was therefore considered to be significantly different to the amount in the noncancerous lung when the *N*_TFPI-2_ value was either >4 (i.e. increased) or <0.25 (i.e. decreased). Such a difference in TFPI-2 gene expression between the tumour and the corresponding noncancerous lung was found in 34 patients (Figure 2A). Indeed, *N*_TFPI-2_ values were at least four times higher in 12 cases (20%) and in contrast significantly lower than 0.25 in 22 patients (37%). In this latter group of patients, the relative decrease in TFPI-2 gene expression varied between patients, ranging between 4- and 120-fold compared to noncancerous lungs (with *N*_TFPI-2_ values varying between 0.25 and 0.008). It was also observed in both stage I–II NSCLC (11/33, 33%) and stage III–IV (11/26, 42%). This downregulation of TFPI-2 gene was observed in both adenocarcinomas (11/35, 31%) and squamous cell carcinomas (9/16, 56%) (Figure 2B). On the other hand, this relative decrease in TFPI-2 mRNA levels was also more frequent in cases of lymph node invasion (48 *vs* 30%) but the difference was not significant (Figure 2C).

### TFPI-2 protein expression in primary lung tumours

Immunostaining for TFPI-2 protein was studied in lung samples of two groups of patients defined according to TFPI-2 gene expression within the tumour. The number of TFPI-2 transcripts within the tumour was higher than 2500 for 10^7^ 18S RNA copies in 11 cases (‘High TFPI-2’ group), while it was lower than 100 in the 11 others (‘Low TFPI-2’ group).

**Figure 3:**
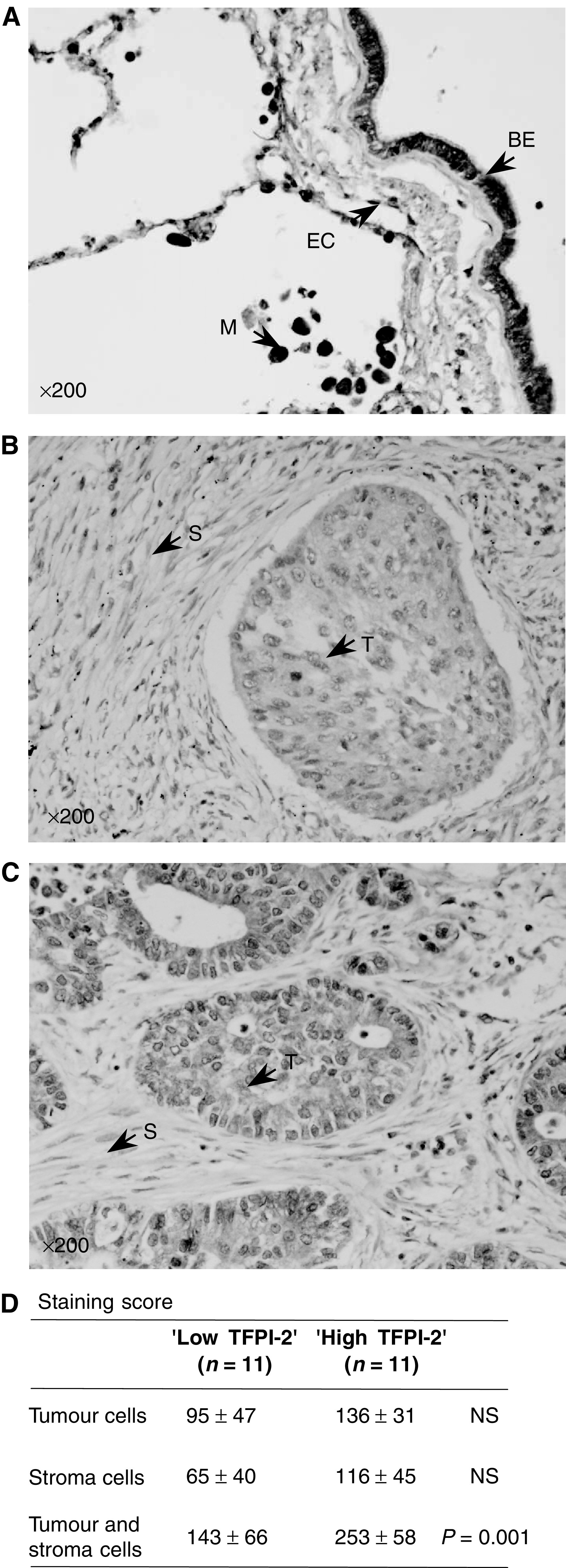
Immunostaining of TFPI-2 in primary tumour and noncancerous tissues. (**A**) Noncancerous lung: TFPI-2 staining of macrophages (M), bronchial epithelium (BE) and endothelial cells (EC). (**B**) Squamous cell carcinoma with no TFPI-2 staining in tumour cells (T) and significant staining in stroma cells (S). (**C**) Adenocarcinoma with significant TFPI-2 staining in tumour cells. Peroxidase technique using polyclonal rabbit antibody specific to human TFPI-2. Haematin counterstaining, original magnification × 200. (**D**) Staining scores in tumour cells and/or stroma cells of NSCLC samples with TFPI-2 mRNA levels <100 (‘low TFPI-2’) or >2500 copies (‘high TFPI-2’). NS=non significant.

TFPI-2 was highly expressed in bronchial epithelium and endothelium cells of noncancerous lungs as well as in alveolar macrophages (Figure 3A). Within tumours, both stroma and cancerous cells appeared to synthesise varying amounts of TFPI-2 protein (Figure 3B–D), and the staining score was well correlated with the number of TFPI-2 mRNA copies measured by real-time PCR (*r*=0.65, *P*<0.01). In addition, the staining scores obtained for the 11 tumours expressing limited amounts of TFPI-2 mRNA were also significantly lower than those of the ‘High TFPI-2’ group (Figure 3D).

**Figure 4:**
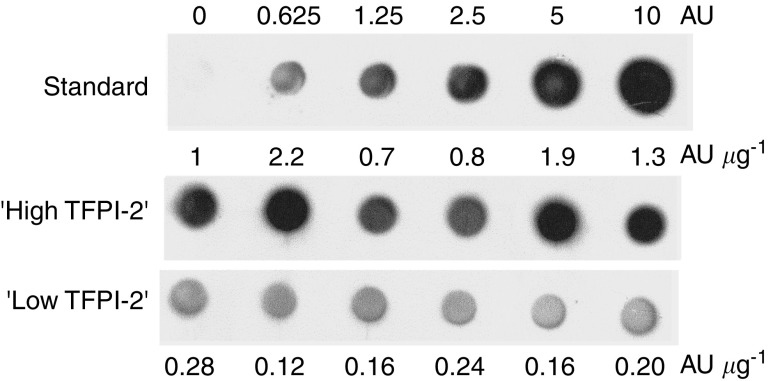
Dot blot analysis. TFPI-2 protein expression in 12 NSCLC with TFPI-2 mRNA level >2500 (‘high TFPI-2’, *n*=6) or <100 copies (‘low TFPI-2’, *n*=6). Results are expressed in AU/*μ*g of total protein according to a standard curve obtained with increasing amounts of proteins from NCI-H23 (line 1). These data are representative of three independent experiments.

Dot blot analysis performed for 12 selected lung tumours also showed that TFPI-2 protein levels were higher in biopsies expressing high levels of TFPI-2 mRNA (range=0.7–2.2 AU *μ*g^−1^ *vs* 0.12–0.28 AU *μ*g^−1^ in ‘low TFPI-2’ tumours, Figure 4).

### Methylation status of promoter TFPI-2 gene in NSCLC

**Figure 5:**
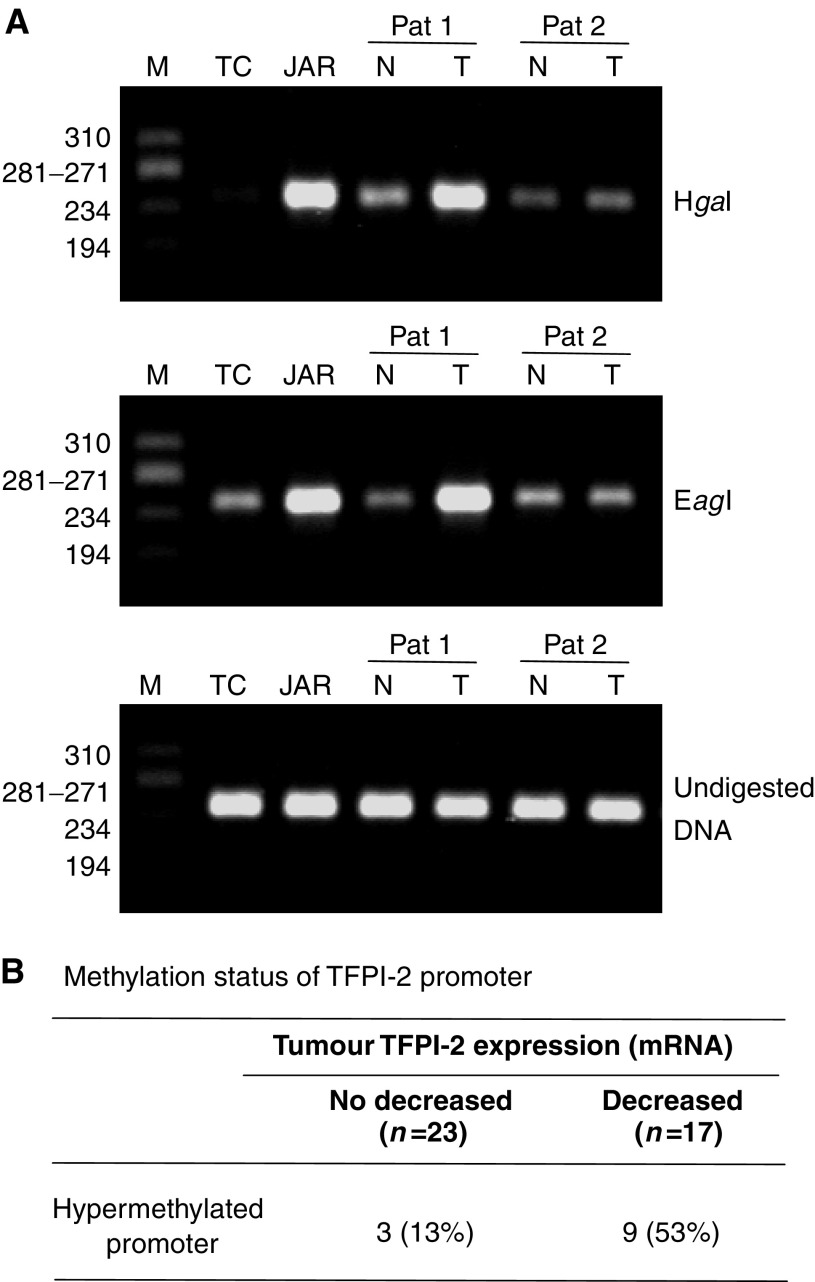
Methylation analysis of TFPI-2 gene promoter in NSCLC using RE-PCR. (**A**) Representative data obtained in two patients are presented (Pat 1: hypermethylated and Pat 2: unmethylated). N=noncancerous tissue; T=lung tumour. JAR=choriocarcinoma cells (i.e. hypermethylated positive control), TC=normal trophoblast cells (i.e. unmethylated control) and M=ϕX174 RF DNA/*Hae*III DNA. Genomic DNA was digested before PCR with *Hgal* or *Eagl*, and results obtained with undigested DNA are also presented. (**B**) Methylation status of TFPI-2 promoter in relation to relative expression of the gene within lung tumours of 40 patients.

The methylation status of TFPI-2 promoter within the tumour was studied by RE-PCR using two different methyl-sensitive restriction enzymes, H*ga*I and E*ag*I. When the TFPI-2 gene promoter was not methylated, such as in normal trophoblast cells, a faint band was obtained after PCR with H*ga*I- or E*ag*I-digested DNA (Figure 5A). In contrast, a strong band was observed when the TFPI-2 promoter was hypermethylated, such as in choriocarcinoma JAR cells chosen in every experiment as positive controls. Band intensity was therefore similar with these cells, whether genomic DNA was treated by H*ga*I or E*ag*I or not. Lung samples of 40 patients with primary NSCLC were studied using this RE-PCR method. In 12 cases (30%), the band intensity after PCR performed following digestion of tumour DNA by H*ga*I and E*ag*I was at least twice as high as the level measured after identical treatment of nonaffected tissue DNA (as illustrated for one case, Pat 1, Figure 5A). In contrast, the band intensities of PCR products were similarly reduced after RE-PCR on tumour and noncancerous tissue DNA in 28 patients compared to the intensity of undigested DNA (see Pat 2, Figure 5A), and this result supported the unmethylated status of TFPI-2 gene promoter in these cases. When these results were compared to those obtained by real-time PCR, the TFPI-2 gene promoter was hypermethylated in nine of the 17 lung tumours (53%) for which gene expression was lower than in noncancerous tissue. In contrast, this epigenetic modification was demonstrated by RE-PCR in only three of the 23 lung tumours (13%) in which no decrease in TFPI-2 mRNA synthesis was found (*P*=0.016, Figure 5B).

**Figure 6:**
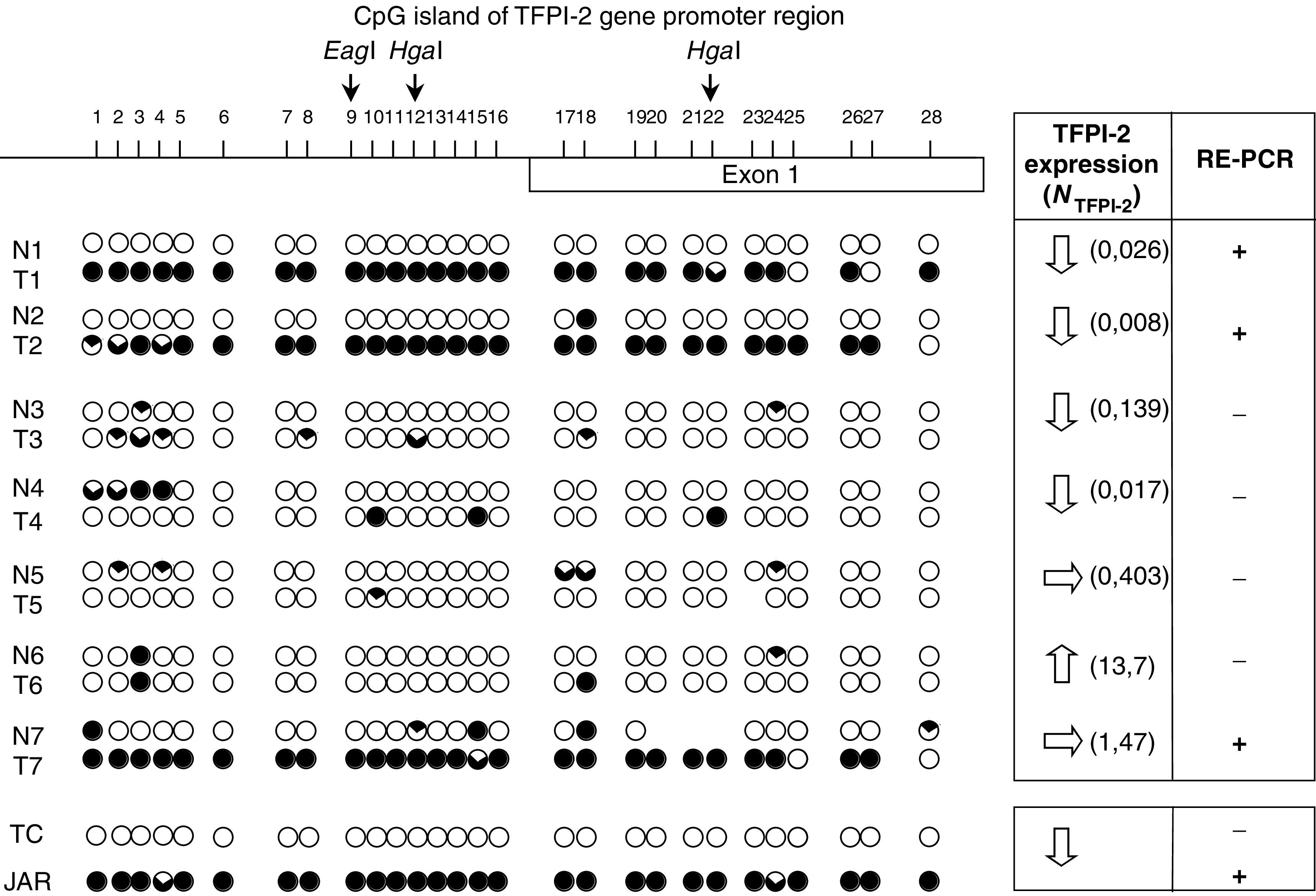
Methylation analysis of TFPI-2 gene promoter in lung cancer and noncancerous tissues using a bisulphite genomic sequencing method. The CpG methylation status of a 250 bp region (−207/+48 from translation start site) of TFPI-2 promoter was studied by the bisulphite genomic sequencing method in seven patients selected according to TFPI-2 expression and results of RE-PCR (N=nonaffected lung, T=tumour). Numbers at the top (1 to 28) correspond to the relative position of CpG sites in this region. Restriction sites for *Eagl* and *Hgal* are also indicated. Each row of circles shows sequencing data obtained from three different clones. Each circle represents a single CpG site that was either nonmethylated (O) or methylated (
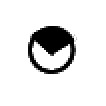
 =one out of three clones, 
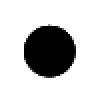
 =two out of three clones, •=three out of three clones).

To validate these results obtained by RE-PCR, bisulphite genomic sequencing was then used to determine the precise methylation status of each of the 28 CpG dinucleotides within a 250 bp region of the TFPI-2 gene promoter in selected samples. Treatment of genomic DNA by sodium bisulphite converts unmethylated cytosines to uracils while 5-methylcytosines remain unmodified. The methylation profile of the TFPI-2 gene promoter sequence could thus be precisely defined by sequencing PCR products obtained from bisulphite-treated DNA. Seven primary NSCLC tumour samples and seven nonaffected biopsies obtained from the same patients were examined using this method, and these samples were chosen according to TFPI-2 gene expression (real-time PCR) and methylation status as evaluated by RE-PCR (Figure 6). For all patients studied, the results of bisulphite genomic sequencing were in accordance with those obtained by the RE-PCR method. In four patients TFPI-2 gene expression was decreased within the tumour compared to nonaffected tissue, and in two of them 26 of 28 CpG dinucleotides were methylated. In contrast, for the two others, the TFPI-2 gene promoter was unmethylated in both the tumour and noncancerous tissues. A similar unmethylated status of the promoter sequence was also demonstrated in two patients for whom TFPI-2 gene expression was not decreased (Pat 5) or increased (Pat 6) within the lung tumour. However, in one case, a high rate of promoter methylation was identified, although TFPI-2 mRNA levels were comparable in nonaffected lung tissue and the tumour.

### TFPI-2 gene expression and promoter methylation compared to clinicopathologic features in NSCLC

No significant difference in TFPI-2 gene expression or promoter methylation was found according to sex, smoking status, or the histological type of lung cancer (Table 2). In addition, the frequency of reduced TFPI-2 gene expression was not statistically different between T1–T2 and T3–T4 lung tumours. In contrast, the TFPI-2 promoter was hypermethylated in eight of 16 patients for whom lymph node involvement was demonstrated, compared to four of 24 with N0 status (*P*=0.02). Moreover, decreased expression of TFPI-2 associated with hypermethylation of the gene promoter was found in eight out of 10 patients with advanced stage of cancer (III or IV) compared to only one out of seven with stage I or II NSCLC (*P*=0.02).

## DISCUSSION

A relative decrease in TFPI-2 mRNA synthesis was demonstrated in this study in 37% of NSCLC using a real-time reverse transcription–PCR (RT–PCR) assay. Real-time PCR is a sensitive and reproducible method that represents a significant advance for the analysis of TFPI-2 gene expression compared to Northern blot analysis (Izumi *et al*, 2000), semiquantitative RT–PCR (Iochmann *et al*, 1999) and competitive RT–PCR (Iochmann *et al*, 2002). In particular, this procedure allowed us to detect significant levels of TFPI-2 mRNA in noncancerous lung tissue, whereas such expression had not been previously demonstrated by RNA blot hybridisation (Izumi *et al*, 2000). Quantification of TFPI-2 mRNA levels using real-time PCR was found to be highly reproducible, with low inter- and intra-assay variations. However, differences in TFPI-2 mRNA levels were recorded when we analysed two biopsies sampled within the same tissue (either nonaffected lung or tumour), although they were never more than four-fold. These differences were probably due to heterogeneity of cell composition from one sample to another since different cells within both noncancerous lung tissue and tumours can synthesise TFPI-2, as shown by immunohistochemistry. A decrease in TFPI-2 mRNA level measured by Northern blotting and *in situ* hybridisation has previously been demonstrated in other cancers, particularly in human gliomas (Rao *et al*, 2001). Moreover, TFPI-2 protein is undetectable by Western blotting in high-grade glioblastomas. TFPI-2 protein synthesis has also recently been studied by immunohistochemical procedures in other different tumours (laryngeal, breast, gastric, colon, pancreatic, renal and endometrial cancer) and was shown to decrease when the degree of malignancy increased (Wojtukiewicz *et al*, 2003). As demonstrated by immunohistochemistry, TFPI-2 is mainly synthesised in noncancerous lung by bronchial epithelial cells and alveolar macrophages. Within tumours, both stroma cells and cancerous cells were shown to express varying amounts of TFPI-2 protein, and a good correlation between the staining score and mRNA levels measured by real-time PCR was found. Dot blot analysis performed on selected samples also confirmed that TFPI-2 protein levels were lower in tumours in which few transcripts of TFPI-2 gene were measured. In a few tumours, TFPI-2 was almost undetectable in cancerous cells, contributing to the lower protein expression measured for samples expressing not many TFPI-2 mRNA copies.

We then specifically focused on the methylation status of the TFPI-2 gene promoter in order to understand why TFPI-2 mRNA levels were decreased in at least one third of NSCLC. We first employed RE-PCR, which is a rapid assay that allowed us to evaluate the methylation status of three CpG sites among the 28 present in the TFPI-2 gene promoter sequence. Hypermethylation of the TFPI-2 gene promoter was thus demonstrated in 12 of the 40 cases of NSCLC that were studied (30%), including nine for whom TFPI-2 gene expression was decreased compared to noncancerous lungs. To validate the results obtained by RE-PCR, we also analysed lung samples from seven selected patients using the bisulphite genomic sequencing method. Despite being time-consuming, this procedure has the advantage of making the analysis of all CpG sites present within the sequence of interest feasible, and confirmed the results obtained by RE-PCR. In addition, concordant results were also obtained after studying noncancerous and cancerous lung samples collected in these seven patients by a methylation-specific PCR method (data not shown). The frequency of TFPI-2 gene promoter methylation (30%) was relatively high compared to frequencies previously published for other methylated genes in primary lung tumours such as MGMT, DAPK, E-cadherin, RASSF1A, TIMP-3 and p16 (Esteller *et al*, 1999; Tang *et al*, 2000; Zochbauer-Muller *et al*, 2002; Yanagawa *et al*, 2003). Silencing of the TFPI-2 gene associated with hypermethylation of the promoter has recently been described in several cancer cell lines derived from choriocarcinoma (Hube *et al*, 2003b), glioma (Konduri *et al*, 2003), fibrosarcoma, breast and prostate cancers (Rao *et al*, 2003). However, this mechanism is probably not the only cause of TFPI-2 gene silencing since hypermethylation of the TFPI-2 promoter was not demonstrated in eight patients in whom TFPI-2 expression within tumours was decreased. Little is known regarding regulation of transcription of the TFPI-2 gene, but it was recently shown that its silencing could be achieved despite the absence of methylation in both exon 1 and sequences upstream from the transcription initiation site (Rao *et al*, 2003). Histone deacetylation is another potential mechanism for TFPI-2 gene silencing in cancer cells since trichostatin A, which inhibits the histone deacetylase, was shown to be effective in inducing TFPI-2 mRNA synthesis in glioma cells (Konduri *et al*, 2003). The TFPI-2 gene has been mapped on chromosome 7q22 (Miyagi *et al*, 1996), and deletions of the 7q region have been described in several malignant tumours (Atkin and Baker, 1993), but this chromosomal abnormality is not a frequent feature of NSCLC. In contrast, gain in 7q has been associated with higher stages of lung tumours and positive nodal involvement on higher tumour grades (Pei *et al*, 2001). On the other hand, as supported by findings obtained with a human fibrosarcoma cell line (HT-1080) and normal human fibroblasts, the RAS oncogene, which can be mutated in NSCLC (Zochbauer-Muller *et al*, 2002), may also contribute to a downregulation of TFPI-2 gene expression (Izumi *et al*, 2000). This hypothesis deserves further study since it was recently shown that regulation of the human TFPI-2 gene is mediated through the Ras/Raf/MEK/ERK pathway (Kast *et al*, 2003). We also found that the TFPI-2 gene promoter sequence was hypermethylated in three of 23 cases of NSCLC although mRNA levels within the tumours were not decreased. Incomplete digestion of DNA-sensitive sites by restriction enzyme is a well-known limitation of RE-PCR (Fraga and Esteller, 2002), but this technique also possibly detected either tumour cells having only one methylated allele or a subfraction of methylated cells within samples. Finally, we also found that TFPI-2 gene promoter was more frequently hypermethylated in patients with lymph node metastases. In addition, decreased TFPI-2 gene expression and promoter hypermethylation were more frequently associated with advanced stages (i.e. III and IV) of lung cancer. During tumour progression, malignant cells may invade adjacent tissues, particularly lymph nodes, and increased plasmin activity in the vicinity of malignant cells, with subsequent activation of MMPs and ECM degradation, enhances tumour invasion and metastases (Rao *et al*, 1999, 2000; Konduri *et al*, 2000). Reduced synthesis in NSCLC of TFPI-2, a potent inhibitor of plasmin, might therefore contribute to tumour invasiveness and metastases *in vivo*. This role of TFPI-2 in cancer invasion was recently supported by injecting fibrosarcoma cell lines expressing active or inactive forms of TFPI-2 in mice (Chand *et al*, 2004). Human TFPI-2 could also regulate tumour angiogenesis by reducing synthesis of the VEGF receptor and affect the expression of several genes involved in oncogenesis, invasion and apoptosis (Chand *et al*, 2004). In addition, TFPI-2 could also influence apoptosis of malignant cells by decreasing the activity of caspases 9 and 3 (Tasiou *et al*, 2001). Whether or not these mechanisms are also regulated within the lung by TFPI-2 has to be evaluated, but our findings strongly suggest that low expression of the TFPI-2 gene by tumour or stroma cells could also favour the development of NSCLC and metastases *in vivo*. On the other hand, the underlying mechanisms for the *de novo* methylation of the TFPI-2 gene in lung cancer cells also remain to be determined. Several mechanisms have been proposed (Tycko, 2000), including overexpression of DNA methyltransferases, increased methylation secondary to overexpression of transcription repressors or loss of transcriptional factors, and intra-allelic transfer of methylation via gene pairing, but all these hypotheses need to be further studied in lung cancer. Silencing of tumour suppressor genes by hypermethylation is a major epigenetic change that contributes to tumour progression. The results obtained in this study indicate that this process can be involved in the downregulation of TFPI-2 expression during NSCLC.

## Figures and Tables

**Table 1:** Oligonucleotide primers used in this study

**Gene**	**Sequences (5′ → 3′)**	**Products PCR (bp)**
TFPI-2 (q)	Forward: AACGCCAACAATTTCTACACCT	125
	Reverse: TACTTTTCTGTGGACCCCTCAC	
18 s (q)	Forward: CGCGGTTCTATTTTGTTGGTTT	120
	Reverse: TTCGCTCTGGTCCGTCTTG	
TFPI-2 (m)	Forward: ACAGTCCCCGTGCATGAATCAGCCAC	238
	Reverse: AGTGCAGCCTCCGTCAGGAAAAGCAGC	

q: primers used for quantitative PCR; m: primers used for methylation.

**Table 2:**
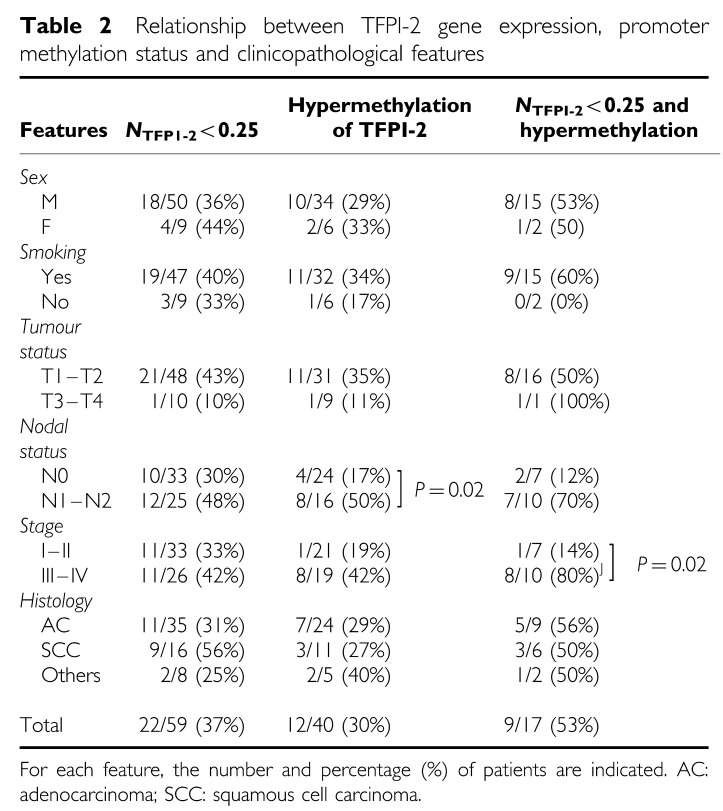
Relationship between TFPI-2 gene expression, promoter methylation status and clinicopathological features
